# Immune Depletion in Combination with Allogeneic Islets Permanently Restores Tolerance to Self-Antigens in Diabetic NOD Mice

**DOI:** 10.1371/journal.pone.0142318

**Published:** 2015-11-18

**Authors:** Nicola Gagliani, Tatiana Jofra, Amanda L. Posgai, Mark A. Atkinson, Manuela Battaglia

**Affiliations:** 1 Diabetes Research Institute (DRI), IRCCS San Raffaele Scientific Institute, Milan, Italy; 2 Vita-Salute San Raffaele University, Milan, Italy; 3 Department of Pathology, Immunology, and Laboratory Medicine, College of Medicine, University of Florida, Gainesville, FL, 32610, United States of America; Baylor College of Medicine, UNITED STATES

## Abstract

The destruction of beta cells in type 1 diabetes (T1D) results in loss of insulin production and glucose homeostasis. Treatment of non-obese diabetic (NOD) mice with immune-depleting/modulating agents (*e*.*g*., anti-CD3, murine anti-thymocyte-globulin (mATG)) can lead to diabetes reversal. However, for preclinical studies with these and other agents seeking to reverse disease at onset, the necessity for exogenous insulin administration is debated. Spontaneously diabetic NOD mice were treated with a short-course of mATG and insulin provided as drug therapy or by way of allogeneic islet implants. Herein we demonstrate that exogenous insulin administration is required to achieve disease reversal with mATG in NOD mice. Unexpectedly, we also observed that provision of insulin by way of allogeneic islet implantation in combination with mATG leads to a pronounced reversal of diabetes as well as restoration of tolerance to self-islets. Expansion/induction of regulatory cells was observed in NOD mice stably cured with mATG and allogeneic islets. These data suggest that transient provision of allogeneic insulin-producing islets might provide a temporary window for immune depletion to be more effective and instilling stable tolerance to endogenous beta cells. These findings support the use of a never before explored approach for preserving beta cell function in patients with recent onset T1D.

## Introduction

Type 1 diabetes (T1D) develops as a result of endogenous insulin insufficiency due to a destruction of the insulin producing pancreatic beta cells [1]. The long held dogma ascribing a 85–90% loss of beta cell mass at disease onset has been recently challenged, and it is now conceivable that sufficient beta cell mass exists near the time of symptomatic onset that, if subjected to the appropriate intervention, might allow for preservation of clinically meaningful metabolic function [1]. A limited number of studies in humans have provided evidence consistent with this notion.

In terms of preclinical efforts, interventions with agents aimed at controlling the ongoing autoimmune response (e.g., anti-CD3, murine anti-thymocyte globulin (mATG)) have been shown to revert diabetes in the NOD mouse model (reviewed in 2). Such reversal efficiency can, depending on agent selection, be significantly improved upon combination with agents intended at repair/regeneration of beta cells (e.g., exendin-4, GLP-1) or targeting the development of regulatory mechanisms [2]. Indeed, we previously reported that low dose mATG in combination with granulocyte colony stimulating factor (G-CSF) treatment leads to durable reversal of diabetes in NOD mice [3] and, more recently, that the same therapy preserves beta cell function in patients with established T1D [4]. However, protocols utilizing NOD mice for disease reversal studies are often not standardized [5] and, as a result, it is still debated whether administration of insulin at the time of diagnosis and shortly thereafter (i.e., two to four weeks) is fundamental for the success of such a therapeutic approach or, on the contrary, it provides further activation of autoreactive immune cells.

The aim of this work was to test this issue by providing insulin either as drug therapy or by way of allogeneic islet implantation. Rationale for this latter option was that allogeneic islets implanted in diabetic NOD mice, along with provision of a new source of “physiological” insulin, would also “divert the attention” of autoreactive immune cells from self-islets to those implanted. This approach, in combination with an immune-targeting therapy, should provide release to the endogenous pancreas and a window for successful therapeutic intervention.

## Materials and Methods

### Animals

NOD/Ltj and Balb/c female mice were purchased from Charles River (Calco, Italy). All mice were maintained under specific pathogen-free conditions. Blood glucose was measured in the morning using an Ascensia Breeze 2 Glucose Meter (Bayer, Leverkusen, Germany). Diabetes was diagnosed after 2 consecutive glucose measurements above 250 mg/dl. This study was carried out in strict accordance with the recommendations in the Guide for the Care and Use of Laboratory Animals of the National Institutes of Health. The protocol was approved by the San Raffaele Hospital Institutional Animal Care and Use Committee (Permit Number: IACUC350). All surgery was performed under isoflurane anesthesia, and all efforts were made to minimize suffering. Mice were sacrificed when blood glucose levels were above 250 mg/dl for 2 consecutive measurements and animals were manifestly suffering (lethargic and motionless) or when blood glucose levels were above 250 mg/dl for 2 consecutive measurements and animals were not manifestly suffering but weight loss was more than 20% (as compared to study entry) [6].

### Treatments

Murine ATG (mATG) was prepared by immunizing rabbits with pooled thymus cells (Genzyme Corporation) and was administered to diabetic NOD mice via two intraperitoneal (IP) injections of 500 μg provided 72 h apart, for a total dose of 1 μg. Some NOD animals received a subcutaneous LinBit insulin implant (LinShin, Scarborough, ON, Canada), providing sustained release of insulin for approximately 2–3 weeks [3]. Other NOD mice received allogeneic islets purified from Balb/c mice and were implanted under the kidney capsule as previously described [7]. Some mice underwent kidney removal as previously described [7]. Finally, a group of NOD mice were treated, at 12 weeks of age, with 250 mg/kg streptozotocin (Sigma, St. Louis, MO) via IP injection, which is known to cause total beta cell ablation. Failure of the therapy was defined as blood glucose levels >250 mg/dl for 2 consecutive measurements. Hyperglycemic NOD mice either died spontaneously or were sacrificed when weight loss was higher than 20% [6].

### Cell staining and enzyme-linked immunosorbent assay (ELISA)

Surface cell staining was performed with anti-CD45 PercP, anti-CD3 APC, anti-CD4 Pacific Blue, and anti-CD25 APC or PeCy7 (all from BD Pharmingen). Foxp3 expression was tested with the Foxp3 staining kit (eBioscience, San Diego, CA; clone FjK-16s). Total pancreatic lymph-node cells (PLN) were cultured for 96 h with anti-CD3 and anti-CD28 mAbs (BD Biosciences, San Jose, CA). Supernatants were collected and IL-10 quantified by a sandwich ELISA using standard commercially available kits (BD Biosciences).

### Statistical analyses

Statistical analyses were performed using GraphPad Prism 5.0 software (GraphPad Software, La Jolla, CA). One-way ANOVA, unpaired t test with two-tailed testing, Kaplan-Meier survival curves and the log-rank test were used. All data are presented as means ± SEM, with P values < 0.05 considered significant.

## Results

**Fig 1:**
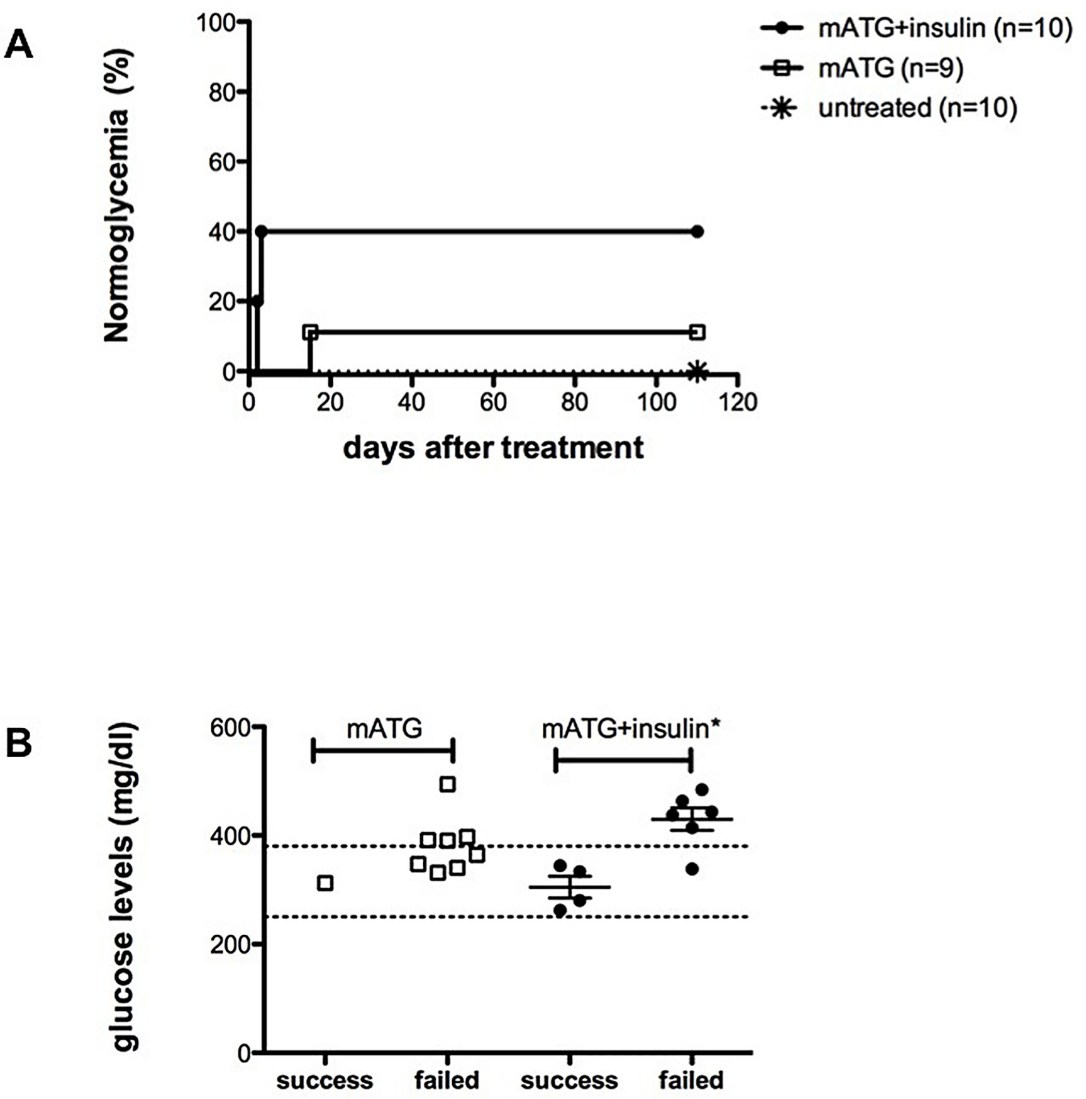
(A) NOD mice with two consecutive blood glucose screenings > 250 mg/dl were treated with mATG + insulin pellet (filled circles, n = 10), or mATG (empty squares, n = 9), or left untreated (asterisks, n = 10). Kaplan-Meyer curves where the endpoint was stable normal glycemia are shown (log-rank test, P = 0.047). (B) Blood glucose levels at study entry (i.e., the day the first mATG dose was administered) are shown per each treated mouse and are divided based on endpoint reached (i.e., success–stable normal glycemia achieved) or not reached (i.e., failed—stable normal glycemia not achieved) (*unpaired t-test, *P* = 0.034). None of the animals used in these experiments died spontaneously.

Administration of mATG in combination with a subcutaneously implanted insulin pellet to spontaneously diabetic NOD mice resulted in durable remission from overt hyperglycemia in 40% of treated mice (4 out of 10), confirming our previous findings [3]. The same reversal effect was not reached in the absence of insulin pellet (Fig 1A), which provides sustained insulin release for approximately 2–3 weeks from implantation [3]. The failure of mATG in curing diabetes in the absence of insulin co-administration did not appear to correlate with blood glucose levels at the time of therapy, yet we confirmed that the therapeutic success of mATG and insulin was largely limited to starting blood glucose values of 380 mg/dl and below (Fig 1B) [3]. This demonstrates that, in diabetic NOD mice, the therapeutic effectiveness of mATG is not reached in the absence of concomitant insulin administration.

**Fig 2:**
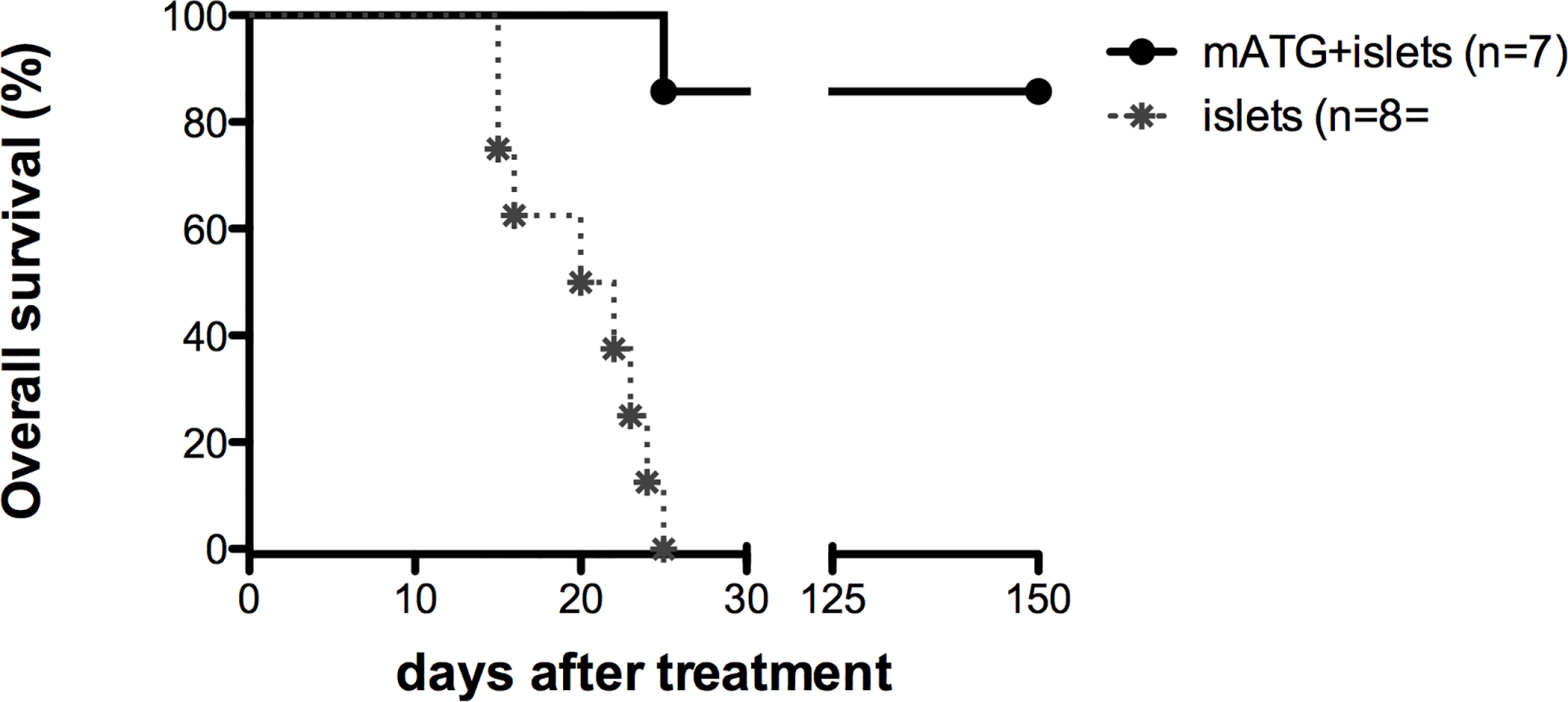
Spontaneously diabetic NOD mice were treated with mATG + allogeneic islets (filled circles, n = 7), or allogeneic islets only (asterisks, n = 8). Kaplan-Meyer curves where the endpoint was death/sacrifice are shown (log-rank test, P = 0.006). Mice died spontaneously of hyperglycemia (n = 3) or were sacrificed when weight loss was higher than 20% (n = 6).

An alternative and theoretically better-controlled release of insulin was considered to be the implantation of purified functional pancreatic islets. Thus, studies were also performed where the insulin pellet was replaced by allogeneic purified islets implanted under the kidney capsule of diabetic NOD mice with starting blood glucose levels between 250 and 380 mg/dl. Allogeneic islets were used rather than those syngeneic for two distinct reasons: (i) in a desire of translating data generated in murine models into humans the possibility to use autologous islets in patients with T1D is, at this moment, extremely scanty; and (ii) allogeneic islets can serve as a source of non-self antigens that would, at least transiently, divert the ongoing immune response from self-islets to those implanted. Thus, diabetic NOD mice received islets from Balb/c animals alone or in combination with mATG treatment. Allogeneic islets and mATG therapy led to an overall survival rate of 85% (6 out 7 animals) while, as expected, none of the mice treated with allogeneic islets alone survived longer than 25 days post onset, despite the two groups having similar blood glucose levels at diabetes onset (Fig 2).

**Fig 3:**
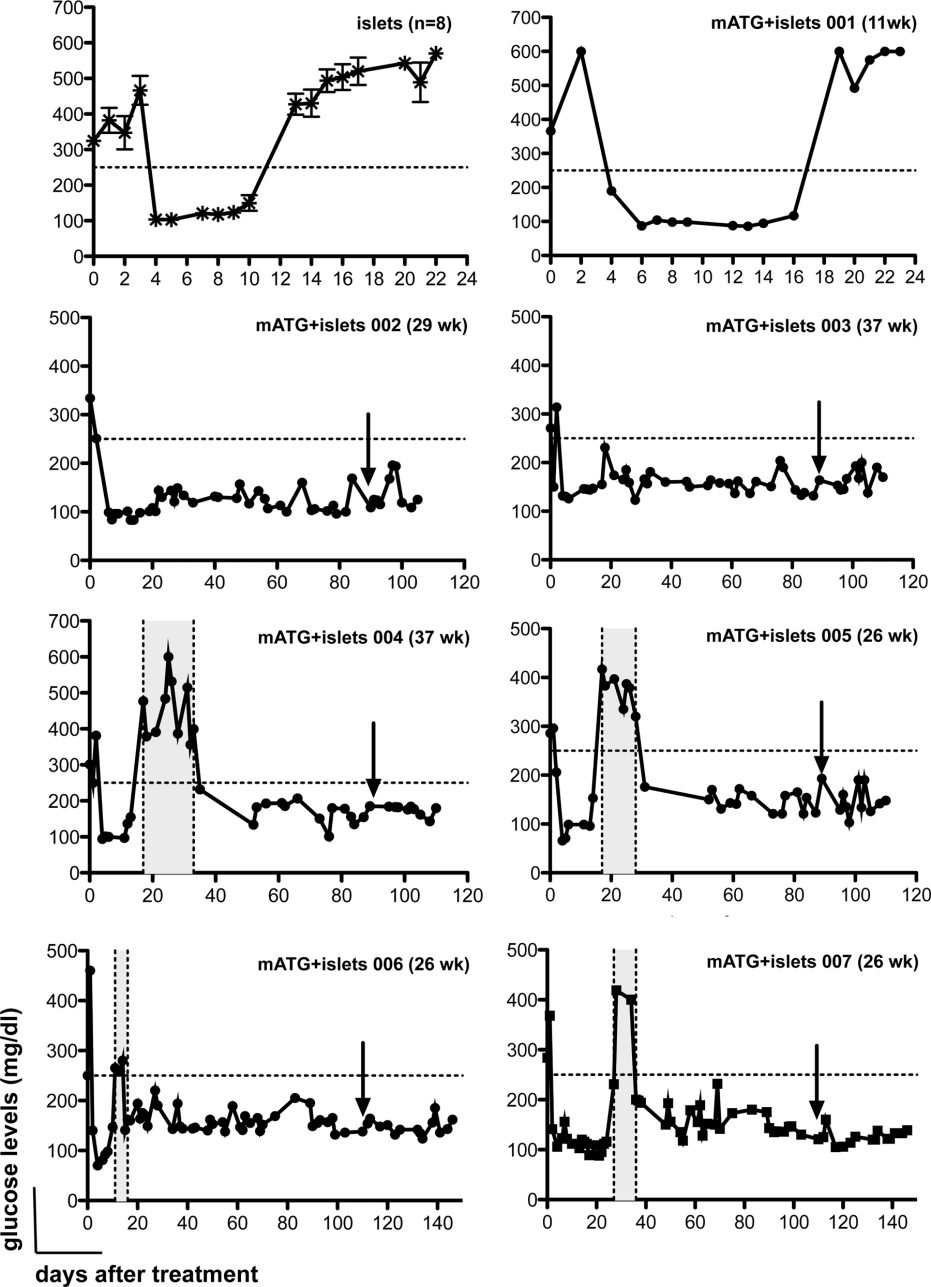
Blood glucose levels of diabetic NOD mice shown in Fig 2 are presented grouped (islets, n = 8—upper left) or individually (mATG+islets mouse 001→007). The age (in weeks, wk) at which the mice entered the study is also reported as median±SD for islets treated animals and individually for mATG+islets treated animals. Horizontal dashed lines indicate blood glucose level above which mice are considered diabetic (i.e., 250 mg/dl). Vertical dashed lines filled with grey area indicate the time of transient hyperglycemia post-treatment. Arrows indicate the day of nephrectomy. Blood glucose levels are reported from the day of study entry until sacrifice (when weight loss was more than 20%) or upon spontaneous death for hyperglycemia.

Interestingly, steady normoglycemia in allogeneic islets and mATG-treated NOD mice was not provided by tolerated implanted allogeneic islets but rather by syngeneic islets released from the burden of autoimmunity. Indeed, all NOD mice cured of diabetes by mATG and islet therapy restored steady normoglycemia 23 days after treatment (mean ± 7.3 SEM) and remained normoglycemic even upon removal of the kidney containing the implanted allogeneic islets. This was proven by a strict time course analysis of glucose levels following diabetes onset, treatment and nephrectomy (Fig 3). Of note, 4 out of 6 NOD mice successfully cured of diabetes by mATG and islets therapy returned hyperglycemic at 18 days post treatment (mean ± 3.3 SEM) but only for a limited time of 9 days (mean ± 2.8 SEM) (Fig 3 –mice 004→007). Following this short hyperglycemic state, all mice eventually returned to stable normoglycemia. These data suggest that rejection of allogeneic islets occurs in all allogeneic islets and mATG-treated mice, in some with a manifest but transient hyperglycemic state, consequently providing for stable healing of endogenous islet function.

**Fig 4:**
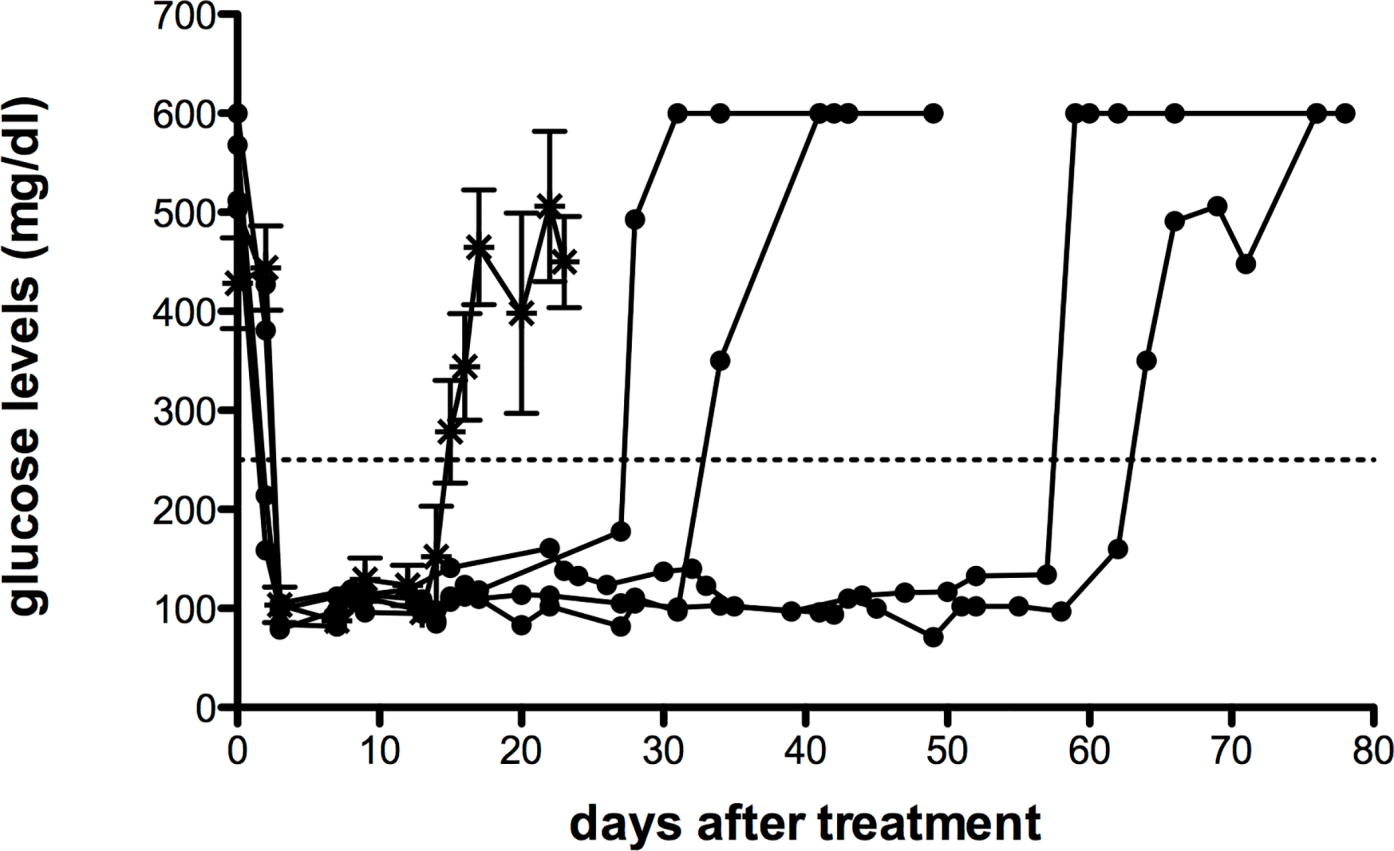
NOD mice were treated with streptozotocin (STZ) at 12 weeks of age. Diabetic STZ-NOD mice were treated with mATG + allogeneic islets (filled circle, individual animals are shown n = 4), or allogeneic islets (asterisk, n = 5 mice are grouped and mean±SEM values are shown per each time point). Horizontal dashed lines indicate blood glucose level above which mice are considered diabetic (i.e., 250 mg/dl). Blood glucose levels are reported from the day of study entry until sacrifice (when weight loss was more than 20%) or upon spontaneous death for hyperglycemia (n = 2).

To further prove endogenous islet recovery upon allogeneic islets and mATG therapy, the exact same treatment was administered to NOD mice turned diabetic upon administration of streptozotocin (STZ) at 12 weeks of age when still normoglycemic. STZ-induced diabetic NOD mice were implanted with allogeneic islets and treated (or not) with mATG. All STZ-treated diabetic NOD mice promptly returned normoglycemic upon islet implantation. Nevertheless they all resumed hyperglycemia. Mice treated with mATG and islets returned to hyperglycemia with a significant delay (46 days±8.9; mean±SEM) in comparison to those receiving only allogeneic islets (17 days±1.4; mean±SEM). However, none of the STZ-induced diabetic NOD mice treated with mATG and islets remained stably normoglycemic (Fig 4).

**Fig 5:**
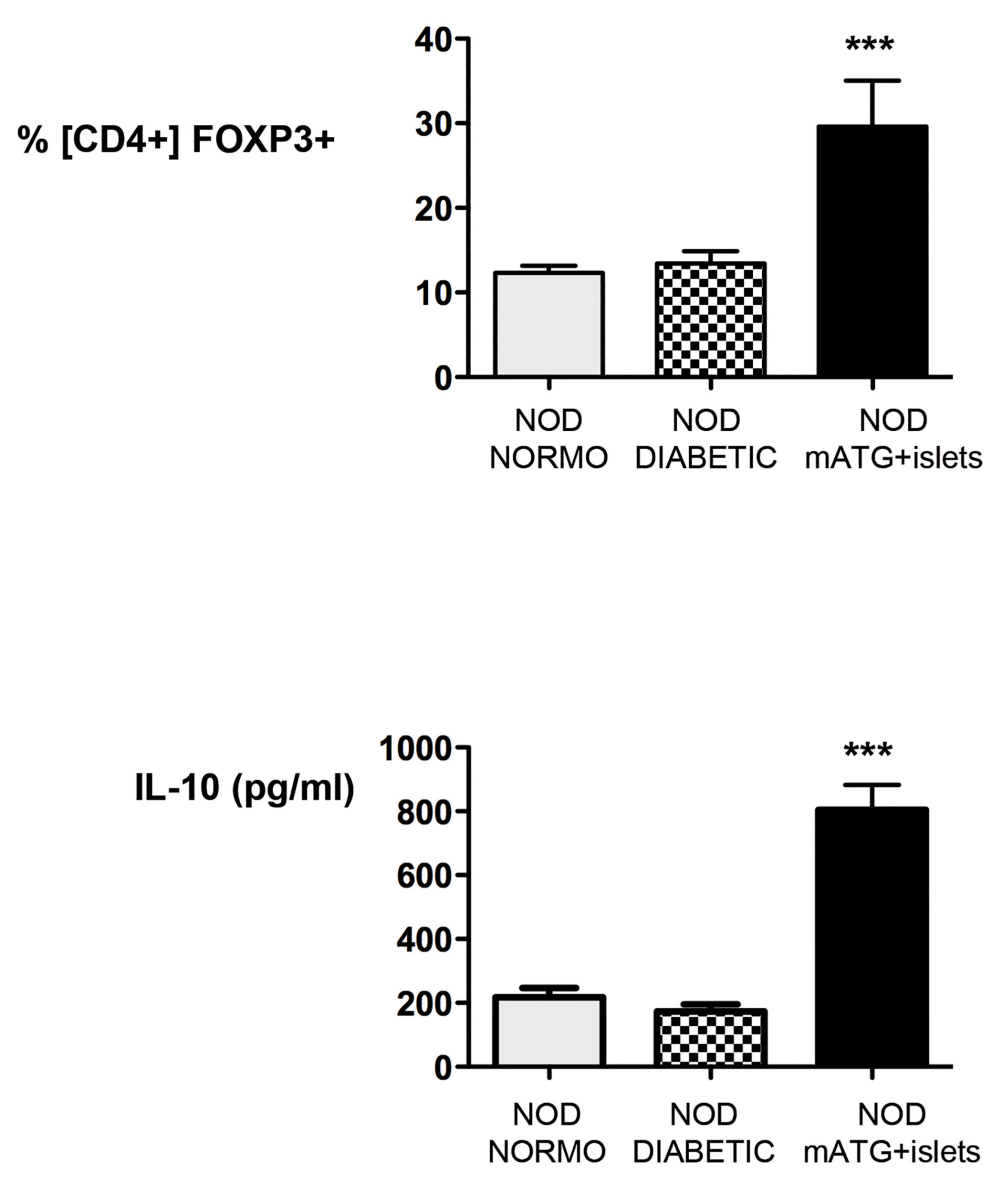
NOD mice shown in Fig 3 (mATG+islets 002, 005, 007) were sacrificed at the end of the study and pancreatic lymph-nodes collected (black bars, n = 3). In parallel, normoglycemic NOD mice (10 week olds, grey bars, n = 2) and diabetic NOD mice (20 week olds, squared bars, n = 3) were sacrificed and used as controls. The frequency of CD4^+^Foxp3^+^ T cells was tested by flow cytometry (upper panel) and the amount of IL-10 released by total pancreatic lymph-node cells upon polyclonal activation was measured by ELISA (lower panel) (mean±SEM are shown and One-way ANOVA, *** P≤0.0005).

To test whether allogeneic islets and mATG therapy in spontaneously diabetic NOD mice contributes to restored tolerance to self-islets via induction of immune-regulation, regulatory cells were tested in the pancreas draining lymph-nodes (PLN) and in the spleen of stably reversed NOD mice (*i*.*e*., 26 days±5.5 normoglycemic after nephrectomy; mean±SEM). CD4^+^Foxp3^+^ T cells and IL-10 producing cells were found significantly expanded in PLN, and not in the spleen, of treated mice thus suggesting the induction of active regulatory mechanisms still detectable more than 100 days after treatment (Fig 5).

## Discussion

This study shows that provision of allogeneic islets to spontaneously diabetic NOD mice, having some levels of preserved islet mass and/or function, in combination with immune depletion, leads to restored tolerance to self-islets upon rejection of those of allogeneic origin. CD4^+^Foxp3^+^ Treg cells and IL-10–producing cells are found expanded/induced in the pancreatic draining lymph-nodes of stable normoglycemic animals, even over 100 days after treatment.

The existence of a sufficient beta cell mass that can recover near the time of symptomatic onset is now accepted [8], and our data further corroborate this notion in NOD mice. In addition to that, our results now suggest that, even in the absence of an islet-regenerative therapy (e.g., GLP-1 or exendin-4) [9] or an additional immunomodulatory treatment (e.g., G-CSF) [3], mATG combined with allogeneic islets leads to restored tolerance to self-islets, likely via regulatory-mediated mechanisms. Immune resetting with mATG is required since implantation of allogeneic islets alone did not restore tolerance to self-antigens.

It has already been demonstrated that mATG can be tolerogenic [10,11], but our previous study showed that mATG + insulin pellet had a low reversal capacity in vivo and the frequency of Tregs were not changed compared to untreated control NOD mice. In contrast, the addition of G-CSF was needed to improve reversal rates and to lead to expansion/generation of CD4^+^CD25^+^FoxP3^+^ Tregs [3]. This suggests that the concomitant administration of allogeneic islets might somehow potentiate the mATG activity in vivo. Alternatively, differences in the results might be due to variances in the time points of analysis between these two studies (i.e., 60 days after treatment in our previous work [3] and more than 100 in our current study).

Taken together, our data provide a fertile ground for considering several mechanistic hypotheses, ones not mutually exclusive. Namely: (i) hyperglycemia observed in diabetic NOD mice may not be due to beta-cell death but rather to deterioration of beta-cell function with consequent failure in insulin release (potentially recoverable); (ii) allogeneic islets implanted under the kidney capsule provides glucose homeostasis (for at least 10/15 days after implant even in the absence of any treatment); (iii) immune cells are likely deviated from the pancreas to the kidney where allogeneic islets have been implanted and a new fresh inflammation occurs, allogeneic antigens are presented, and relevant self-antigens have been reintroduced in a dangerous environment; (iv) endogenous beta-cells are metabolically relieved and re-start producing insulin. If no therapy is provided, once the immune system rejects the newly implanted allogeneic islets, it returns to be active against the pancreas. On the contrary, in the presence of immune-modulating therapy, such as mATG, the kidney-attracted immune cells (both self- and allo-reactive) are controlled and active mechanisms of regulation (such as CD4^+^Foxp3^+^ Tregs and IL-10 producing cells) are also generated. This re-balanced immune system is no longer more harmful to the pancreas, which has recovered close to its normality. Alternatively, immune cells may remain in the pancreas while new Tregs are generated in the transplant, where new antigens are released and are then, attracted to the pancreas, mediating active immune-regulation and self-tolerance restoration. We also cannot exclude that graft-derived factors play a key role in modulating immune cells, thus leading to effector cell deletion or immune deviation. To be clear, formal proofs for each of these hypotheses are still lacking and need be the subject of future investigation.

While our study provides for a series of new hypotheses, we must emphasize its limitations. First, at this point we cannot exclude that allogeneic islets, in addition to restoring euglycemia via insulin release, are also contributing to the observed immune-deviation via release of other factors. Second, to prove that allogeneic islets “attract” self-reactive cells and thus distract the immune system from self-attack one should administer to diabetic animals any other cell type of allogeneic origin in combination with a euglycemia-restoring treatment. In addition, in the absence of histological analysis of tissues (e.g., pancreas and transplanted islets) it is impossible to know the processes that influenced disease outcomes within the target organs. Lastly, although the tolerogenic markers that were tested (IL-10 production and Foxp3^+^ T cells) are suggestive of a Treg-mediated mechanism, formal proof of an active regulatory process is still lacking. However, our previous experiences in the same animal model are highly supporting of such a mechanism [12,13].

All in all, these data provide compelling support for the use of a more “aggressive” but transient therapeutic approach in patients with recent-onset T1D with the aim of deviating the immune system from self- to allogeneic-cells thus leaving a temporary window for immune-depletion/modulation to be more effective and for endogenous beta-cells to recover.

## References

[1] Atkinson MA, Eisenbarth GS, Michels AW (2014) Type 1 diabetes. Lancet 383: 69–82. 10.1016/S0140-6736(13)60591-7 23890997 PMC4380133

[2] Roep BO, Tree TI (2014) Immune modulation in humans: implications for type 1 diabetes mellitus. Nat Rev Endocrinol 10: 229–242. 10.1038/nrendo.2014.2 24468651

[3] Parker MJ, Xue S, Alexander JJ, Wasserfall CH, Campbell-Thompson ML, Battaglia M, et al (2009) Immune depletion with cellular mobilization imparts immunoregulation and reverses autoimmune diabetes in nonobese diabetic mice. Diabetes 58: 2277–2284. 10.2337/db09-0557 19628781 PMC2750219

[4] Haller MJ, Gitelman SE, Gottlieb PA, Michels AW, Rosenthal SM, Shuster JJ et al (2015) Anti-thymocyte globulin/G-CSF treatment preserves beta cell function in patients with established type 1 diabetes. J Clin Invest 125: 448–455. 10.1172/JCI78492 25500887 PMC4382237

[5] Atkinson MA (2011) Evaluating preclinical efficacy. Science translational medicine 3: 96cm22 10.1126/scitranslmed.3002757 21849661

[6] Zhao C, Wang Z, Robertson MW, Davies JD (2008) Cachexia in the non-obese diabetic mouse is associated with CD4+ T-cell lymphopenia. Immunology 125: 48–58. 10.1111/j.1365-2567.2008.02819.x 18397274 PMC2526259

[7] Gagliani N, Jofra T, Valle A, Stabilini A, Morsiani C, Gregori S, et al (2013) Transplant tolerance to pancreatic islets is initiated in the graft and sustained in the spleen. Am J Transplant 13: 1963–1975. 10.1111/ajt.12333 23834659 PMC3869180

[8] Akirav E, Kushner JA, Herold KC (2008) Beta-cell mass and type 1 diabetes: going, going, gone? Diabetes 57: 2883–2888. 10.2337/db07-1817 18971435 PMC2570380

[9] Pettus J, Hirsch I, Edelman S (2013) GLP-1 agonists in type 1 diabetes. Clin Immunol 149: 317–323. 10.1016/j.clim.2013.04.006 23643354

[10] Maki T, Ichikawa T, Blanco R, Porter J (1992) Long-term abrogation of autoimmune diabetes in nonobese diabetic mice by immunotherapy with anti-lymphocyte serum. Proc Natl Acad Sci U S A 89: 3434–3438. 1565635 10.1073/pnas.89.8.3434 PMC48882

[11] Mohty M (2007) Mechanisms of action of antithymocyte globulin: T-cell depletion and beyond. Leukemia 21: 1387–1394. 17410187 10.1038/sj.leu.2404683

[12] Battaglia M, Stabilini A, Draghici E, Migliavacca B, Gregori S, Bonifacio E, et al (2006) Induction of tolerance in type 1 diabetes via both CD4+CD25+ T regulatory cells and T regulatory type 1 cells. Diabetes 55: 1571–1580. 16731819 10.2337/db05-1576

[13] Fousteri G, Jofra T, Di Fonte R, Gagliani N, Morsiani C, Stabilini A, et al (2015) Lack of the protein tyrosine phosphatase PTPN22 strengthens transplant tolerance to pancreatic islets in mice. Diabetologia 58: 1319–1328. 10.1007/s00125-015-3540-9 25748328

